# The effect of healthcare disruptions during the COVID‐19 pandemic on colposcopy services and practice: A systematic review and meta‐analysis

**DOI:** 10.1111/aogs.70066

**Published:** 2025-10-08

**Authors:** Giovanni Delli Carpini, Zahid Mammadov, Simon Leeson, Anne Hammer, Mihaela Grigore, Andrea Ciavattini

**Affiliations:** ^1^ Gynecologic Section, Department of Odontostomatologic and Specialized Clinical Sciences Università Politecnica Delle Marche Ancona Italy; ^2^ Northern Gynaecological Oncology Centre Queen Elizabeth Hospital Gateshead UK; ^3^ European Federation for Colposcopy, Triumph Benelux Brussels Belgium; ^4^ Department of Clinical Medicine Aarhus University Aarhus Denmark; ^5^ Department of Obstetrics and Gynecology, Gødstrup Hospital University Clinic for HPV‐Related Gynecological Disease Herning Denmark; ^6^ Department Obstetrics and Gynecology University of Medicine and Pharmacy “Grigore T. Popa” Iasi Romania

**Keywords:** colposcopy, COVID‐19, early detection of cancer, healthcare disparities, uterine cervical neoplasms

## Abstract

**Introduction:**

The healthcare reorganization during the COVID‐19 pandemic affected colposcopy services and cervical cancer prevention, particularly in those countries where healthcare systems were already under‐resourced. This review aimed to quantify the reduction in colposcopy services across countries during the COVID‐19 pandemic and to determine whether the data source per study and cervical cancer screening coverage per country influenced the extent of these reductions.

**Material and Methods:**

Studies reporting comparative data on colposcopy services between the COVID‐19 pre‐pandemic and pandemic period were included. MEDLINE, Embase, EMCare, Covid‐19 Research, British Nursing Index, APA PsycINFO, and Allied and Complimentary Medicine databases were searched for studies published from March 2020 to December 2023. The Newcastle−Ottawa scale was used for risk of bias assessment. The number of colposcopies, cervical treatments, pre‐invasive lesions diagnoses, and cervical cancer diagnoses per month were compared between the pre‐pandemic (before March 2020) and pandemic period (after March 2020). The effect measure was the standardized mean difference. Heterogeneity was evaluated with the chi‐squared test and quantified with the *I* ^2^ method. A meta‐regression was performed, considering the data source (regional/national databases/registries or institutional databases) and the screening coverage according to World Health Organization data (≥70% or <70%) as moderators. The review was registered on PROSPERO (CRD42023447188).

**Results:**

Thirteen studies were included. Twelve were of good/high quality according to the Newcastle−Ottawa scale. The standardized mean difference between the pre‐pandemic and pandemic periods was −1.60 (95% CI −2.49 to −0.72, *p* = 0.004) for colposcopies (4 studies, *I* ^2^ = 60.97%, *p* = 0.075), −1.70 (95% CI −2.50 to −0.90, *p* < 0.001) for cervical treatments (5 studies, *I* ^2^ = 52.92%, *p* = 0.081), −4.61 (95% CI ‐7.90 to −1.33, *p* = 0.006) for pre‐invasive lesion diagnoses (4 studies, *I* ^2^ = 92.45%, p < 0.001), and −0.85 (95% CI −1.52 to −0.19, *p* = 0.012) for cervical cancer diagnoses (9 studies, *I* ^2^ = 71.07%, *p* = 0.002). At meta‐regression, further reductions for cervical treatments and pre‐invasive lesion diagnoses were observed in the case of screening coverage <70%.

**Conclusions:**

During the COVID‐19 pandemic, a reduction in colposcopies, cervical treatments, pre‐invasive lesions diagnoses, and invasive cancer diagnoses was observed. Since a screening coverage of <70% heightened these declines, increasing such coverage could lead to better resilience of cervical cancer prevention services to future crises.

## Graphical abstract

The COVID‐19 pandemic has shown that colposcopy services are vulnerable to a global health crisis. Increasing cervical cancer screening coverage is crucial to mitigate the reduction in colposcopy and cervical cancer prevention services during global health emergencies.

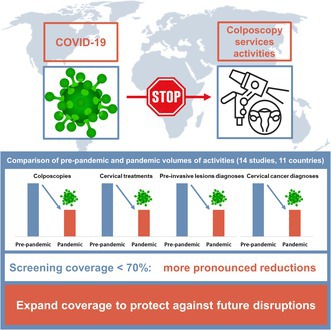

**Abbreviations**
CIN: cervical intraepithelial neoplasia
COVID‐19: Coronavirus disease 2019
SD: standard deviation
SMD: standardized mean difference

**Key message**

The COVID‐19 pandemic has shown that colposcopy services are vulnerable to a global health crisis. Increasing cervical cancer screening coverage is crucial to mitigating the reduction in colposcopy and cervical cancer prevention services during global health emergencies.

## 1 INTRODUCTION

The secondary prevention of cervical cancer relies on a well‐organized and consistently maintained provision of healthcare services. However, this system is highly vulnerable to disruption in the event of large‐scale and unpredictable crises. The COVID‐19 pandemic exemplified this vulnerability, leading to widespread interruptions in cervical cancer prevention programs globally, including colposcopy services, treatment of pre‐invasive lesions, follow‐up after treatment, and the new diagnoses of invasive cancer.^1^, ^2^

The suspension periods for these activities and their subsequent resumptions varied across different regions of the world, resulting in a heterogeneous impact on the volume of services delivered.^1^, ^2^, ^3^, ^4^

For example, although colposcopy services continued receiving referrals, waiting times were affected following a four‐month pause in screening invitations in Wales.^5^ Meanwhile, all non‐urgent health services, including colposcopy, were halted for over 2 months in Canada.^2^ In contrast, cervical cancer prevention services were not suspended in England and Australia.^6^ Even after the resumptions, logistical barriers and patient hesitancy further led to pronounced declines in service volumes. These interruptions disproportionately affected those countries where healthcare systems were already under‐resourced.^3^

Regarding the volume of activities, a survey endorsed by the European Federation for Colposcopy evidenced that 44% of European countries had stopped colposcopies and treatments for cervical precancerous lesions at least once in the period between April 2020 and December 2021.^7^ The reported reductions varied across different categories, ranging from 4.3% to 47% for colposcopies,^8^, ^9^ 15% to 31.1% for cervical treatments,^4^, ^9^ 10% to 16% for the diagnoses of pre‐invasive lesions,^9^, ^10^ and 7% to 74.3% for cervical cancers diagnoses.^3^, ^11^ These differences highlight the disproportionate impact of the pandemic, reflecting disparities in healthcare services, resource availability, and organizational resilience.^1^

The systematic review by Ferrara et al. of 2022 highlighted the significant impact of pandemics on both vaccination and screening services.^12^ Given that colposcopy‐related activities primarily target individuals at increased risk of cervical cancer (e.g., following a positive screening test), it is equally important to examine the effects of the pandemic on these services. Including colposcopy in the assessment provides a more comprehensive understanding of the overall impact of the pandemic on cervical cancer prevention.

This systematic review and meta‐analysis aimed to: (1) provide a comprehensive overview of cervical cancer screening practices during the COVID‐19 pandemic across the countries included in the study; (2) assess the extent of disruptions to screening programs in different settings; (3) quantify the impact of the pandemic on colposcopy services and clinical practice; (4) identify and explore factors contributing to the disproportionate effects observed across various countries and healthcare systems; (5) determine which of these factors may be modifiable, to inform strategies to strengthen resilience and minimize the impact of future public health emergencies on cervical cancer prevention services.

## 2 MATERIAL AND METHODS

We conducted a systematic review according to Preferred Reporting Items for Systematic Reviews and Meta‐Analyses guidelines.^13^ The review was registered with the International Prospective Register of Systematic Reviews (registration number 2023 CRD42023447188) on November 22, 2023.

### 2.1 Data sources

A systematic literature search was conducted using MEDLINE, Embase, EMCare, Covid‐19 Research, British Nursing Index, APA PsycINFO, and Allied and Complimentary Medicine databases. We included only papers written in English and published between March 2020 and December 2023. A gray literature search was carried out via Google Scholar. Additional search strategy information can be found in Supporting File S1.

### 2.2 Main outcomes measures

The population of interest was composed of patients attending colposcopy services after a positive first‐level screening test (cervical cytology or human papillomavirus DNA test), with a histopathological diagnosis of cervical intraepithelial lesion or cervical cancer, in follow‐up after treatment for cervical intraepithelial lesions, or with clinical suspicion of cervical cancer. The exposure was the period of health services disruption during the COVID‐19 pandemic. The volume of colposcopy service activities was compared between the period before the COVID‐19 pandemic (pre‐pandemic period) and the period of health services disruption during the COVID‐19 pandemic (pandemic period). The four primary outcomes were: (1) the number of colposcopies performed in the pathway of cervical cancer prevention; (2) the number of treatments for pre‐invasive lesions, including any treatment modality (loop electrosurgical excision procedure/large loop excision of the transformation zone, carbon dioxide laser conization, cold knife conization); (3) the number of pre‐invasive lesions (cervical intraepithelial neoplasia—CIN2, CIN3, or adenocarcinoma in situ) diagnosed; (4) the number of cervical cancers diagnosed.

### 2.3 Eligibility criteria

Observational studies (case–control, cohort, or cross‐sectional studies) were included according to the following criteria: Inclusion (1) primary reports available in full text; (2) reporting data on colposcopy service activities, diagnostic procedures, and treatments of cervical precancer and cancer; (3) including pre‐ and pandemic data comparison; Exclusion: (1) studies not considering the impact of the COVID‐19 pandemic; (2) narrative review, editorial, or letters to the editor. Studies conducted in both high‐ and low‐income countries were included, without geographic exclusions. The analysis included studies from regions with and without organized cervical screening programs, as well as those with and without human papillomavirus vaccination programs. Two authors (G.D.C. and Z.M.) independently screened the articles obtained from the initial search against inclusion criteria. Disagreements were solved through discussion, and if consensus could not be reached, a third author (S.L.) provided the final judgment.

The Newcastle−Ottawa scale was used for risk of bias assessment.^14^ Studies were rated from 0 to 9, with those studies rated 0–2 considered as “poor quality,” 3–5 as “fair quality,” and “6–9” as good/high quality. Two authors (G.D.C. and Z.M.) performed the assessment independently. Disagreements were solved through discussion; in case of failure of discussion, a different author (S.L.) made the final judgment. The results of the risk of bias assessment were reported both in the manuscript text and summarized in a table.

### 2.4 Data collection and analysis

Two authors (G.D.C. and Z.M.) independently extracted the relevant data from each included study. Disagreements were resolved through discussion; in case of failure of discussion, a different author (S.L.) made the final judgment. The following data were obtained from the included studies: study design, study country, screening coverage (≥70% or <70% according to WHO 2019 data),^15^ primary screening test (according to WHO 2019 data),^15^ data source (regional/national databases/registries or institutional databases), and the time frame definition in months of the pre‐pandemic and pandemic periods. Data regarding the number of colposcopies, the number of pre‐invasive lesions, the number of treatments, and the number of cervical cancer diagnoses were extracted as absolute values per month of both the pre‐pandemic and pandemic periods. Data were retrieved from the manuscripts. For studies reporting only the total number of activities per period and the number of months, the monthly mean per study was obtained by dividing the total count by the number of months. The standard deviation (SD) was estimated as the square root of the mean, assuming a Poisson distribution, which is appropriate for count data.^16^ The standard deviation was directly calculated from the observed values for studies with monthly data available. A pre‐piloted Microsoft Excel spreadsheet was used for data recording.

### 2.5 Statistical analyses

The effect measure for the identified four outcomes was the monthly standardized mean difference (SMD) between the pre‐pandemic and pandemic periods, with a corresponding 95% confidence interval (CI). The following baseline elements were qualitatively synthesized: study design, study country, screening coverage, primary screening test, data source, and the time frame definition of the pre‐pandemic and pandemic period. A meta‐analysis with a random‐effects model was performed for SMD with 95% CI determination for the study outcomes, comparing the pre‐pandemic and pandemic periods. We chose the random effects model due to the expected major heterogeneity in the data across studies. Statistical heterogeneity was evaluated with the chi‐squared test for heterogeneity and quantified with the *I* ^2^ method. Visual display for data presentation was included (forest plot). A funnel plot was generated to evaluate small‐study effects, with the SMD as the effect estimate and the standard error as the measure of precision. The Egger test was used to evaluate funnel plot asymmetry using the weighted regression with the multiplicative dispersion method. A meta‐regression was performed using the variable “data source” (regional/national databases/registries or institutional databases) and the variable “screening coverage” (≥70% or <70%) as dichotomic moderators for the SMD. The RStudio software (version 2022.02.3 Build 492) with the “metafor” package was used.^17^ A p‐value <0.05 was considered statistically significant.

## 3 RESULTS

### 3.1 General characteristics of the studies

**FIGURE 1:**
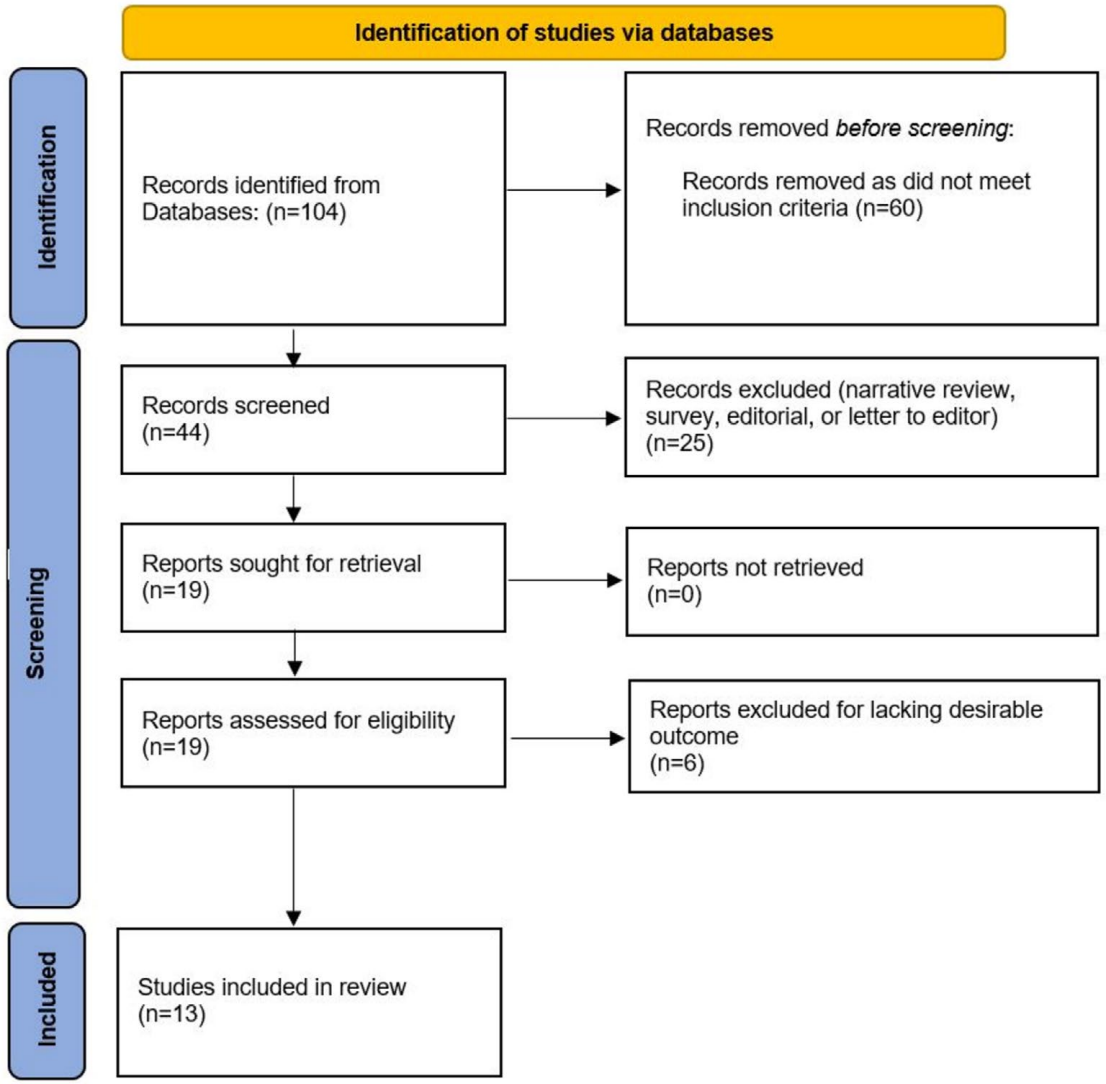
Flow diagram of study selection.

**FIGURE 2:**
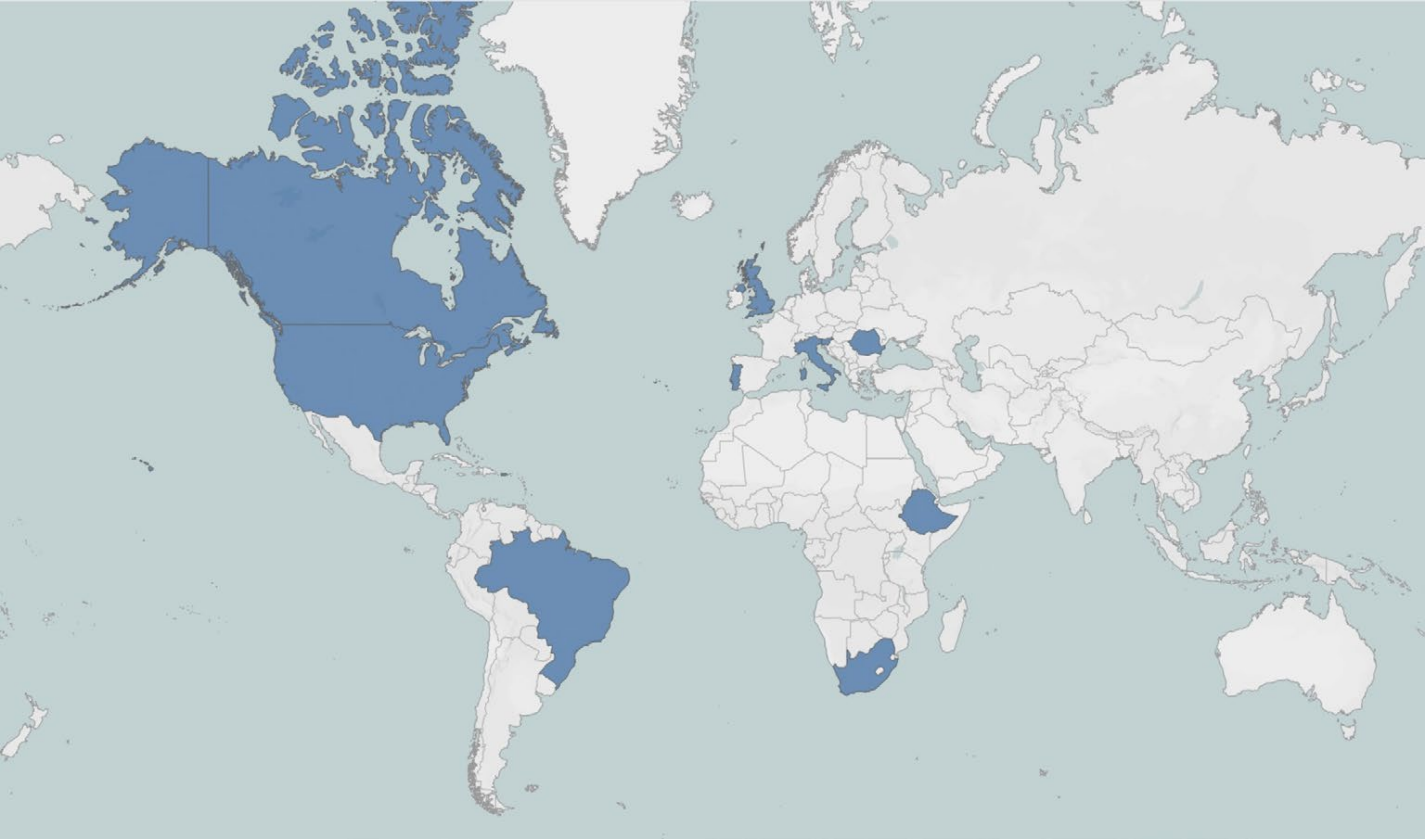
Country origin of the included studies.

**FIGURE 3:**
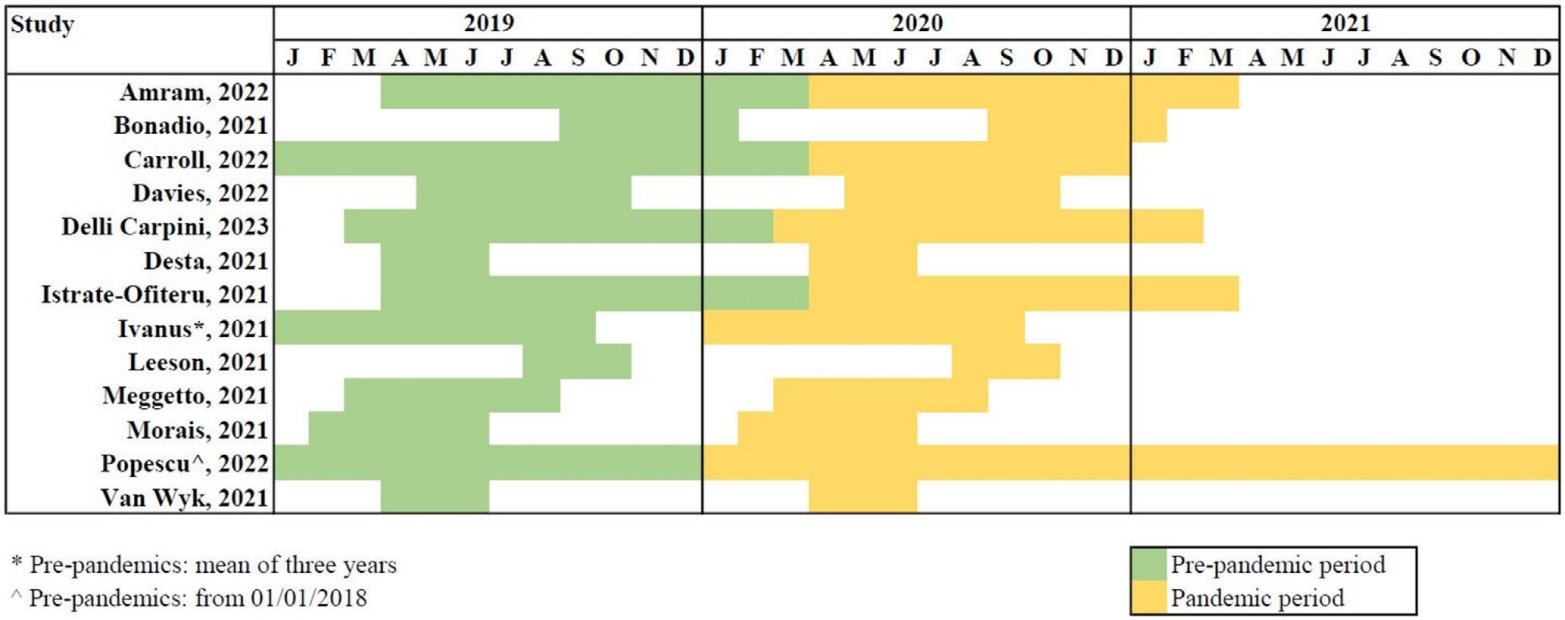
Comparison of pre‐pandemic (green) and pandemic (yellow) periods among the included studies.

We identified 104 studies, 13 of which were included for analysis.^1^, ^3^, ^4^, ^5^, ^8^, ^9^, ^10^, ^11^, ^18^, ^19^, ^20^, ^21^, ^22^ Figure 1 presents the flow diagram of the complete study selection process. The characteristics of each included study are summarized in Table 1. All included studies were retrospective. Six studies (46%) obtained data from a database or registry of cervical cancer screening programs, while the remaining seven (54%) used data from institutional databases. Figure 2 shows the country of origin of each included study. According to WHO data, eight (62%) studies were from countries with a screening coverage ≥70%, and four (30.8%) from countries with HPV test as the primary screening test. Figure 3 compares the time frame definition for the pre‐pandemic and pandemic periods among all included studies. The time frame ranged from 3 to 24 months, with eight (62%) studies comparing the activity volume for at least 6 months.

**TABLE 1:** Characteristics of included studies.

First author	Year	Country	Screening coverage	Primary screening test	Data source	No. of months Pre‐pandemic	No. of months Pandemic
Amram	2022	USA	82%	Cytology	Regional/National registries	12	12
Bonadio	2021	Brazil	41%	Cytology	Institutional databases	5	5
Carroll	2022	USA	82%	Cytology	Regional/National registries	15	9
Davies	2022	England	78%	HPV test	Institutional databases	6	6
Delli Carpini	2023	Italy	87%	HPV test	Institutional databases	12	12
Desta	2021	Ethiopia	3%	VIA	Regional/National registries	3	3
Istrate‐Ofiteru	2021	Romania	38%	Cytology	Institutional databases	12	12
Ivanus	2021	Slovenia	88%	Cytology	Regional/National registries	9	9
Leeson	2021	Wales	78%	HPV test	Regional/National registries	3	3
Meggetto	2021	Canada	87%	Cytology	Regional/National registries	6	6
Morais	2021	Portugal	84%	HPV test	Institutional databases	5	5
Popescu	2022	Romania	38%	Cytology	Institutional databases	24	24
Van Wyk	2021	South Africa	43%	Cytology	Institutional databases	3	3

According to the risk of bias evaluation, one (8%) of the included studies was of “fair” quality, while the remaining 12 (92%) were of “good/high” quality, with two (15%) studies scoring nine points on the Newcastle−Ottawa scale. The complete risk of bias evaluation for each study is reported in Table 2.

**TABLE 2:** Risk of bias evaluation of the included studies.

Study	Selection	Comparability	Outcome	Total
Amram 2022	****	—	***	7
Bonadio 2021	****	—	***	7
Carroll 2022	****	**	***	9
Davies 2022	****	—	***	7
Delli Carpini 2023	****	—	***	7
Desta 2021	****	—	***	7
Istrate‐Ofiteru 2021	****	—	***	7
Ivanus 2021	****	—	***	7
Leeson 2021	****	—	***	7
Meggetto 2021	****	—	**	6
Morais 2021	****	**	***	9
Popescu 2022	****	—	***	7
Van Wyk 2021	****	—	***	7

* = one point; — = 0 points.

### 3.2 Synthesis of the results

**FIGURE 4:**
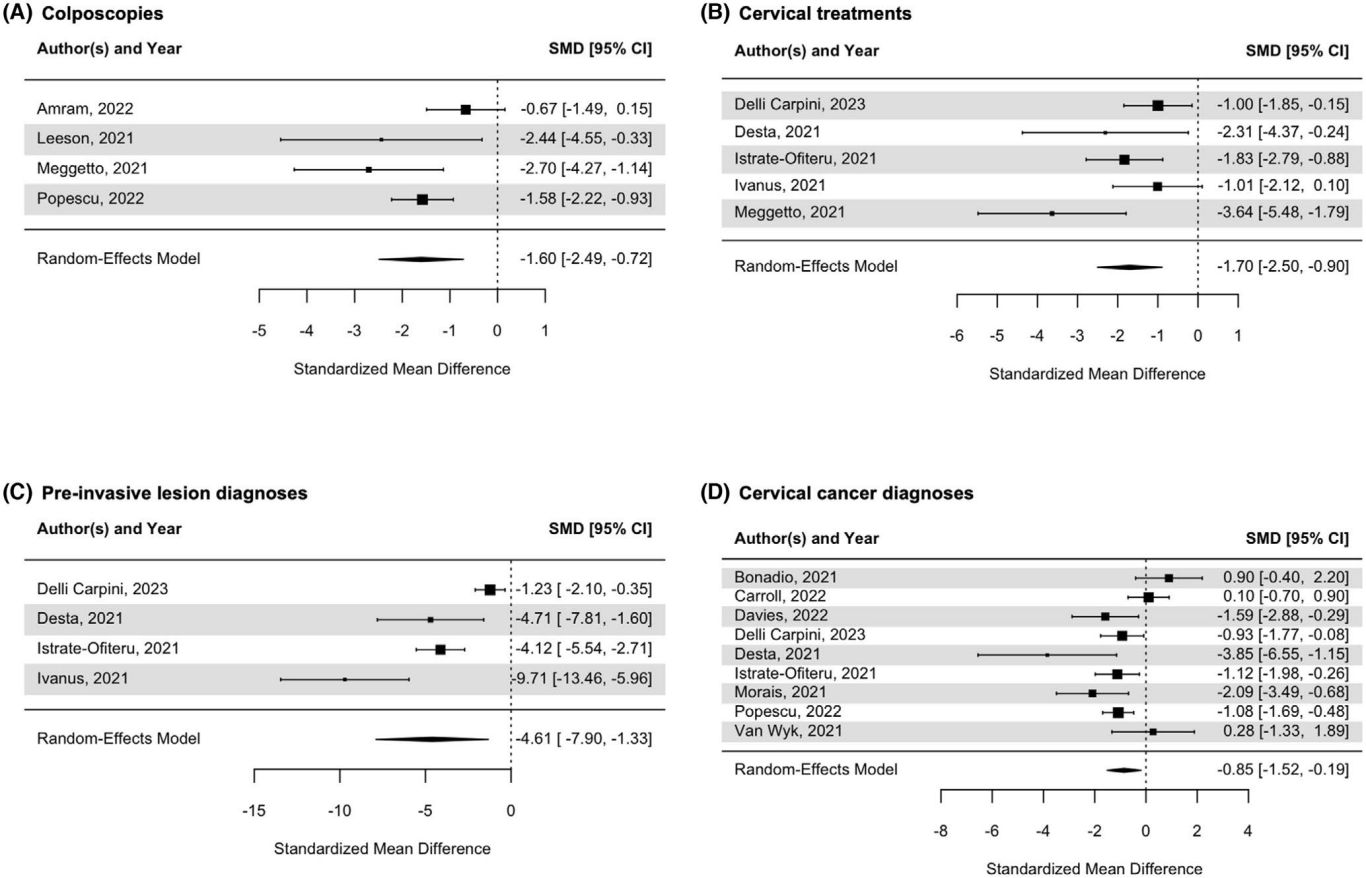
Forest plot for standardized mean difference of review outcomes.

The reported number of colposcopies ranged from 108 to 51 684 in the pre‐pandemic period and from 41 to 30 356 in the pandemic period.^1^, ^4^, ^5^, ^8^ In the pandemic period, the number of colposcopies per month was reduced by −1.60 SD (95% CI −2.49 to −0.72, *p* = 0.004) (Figure 4A). The reported number of cervical treatments ranged from 20 to 4419 in the pre‐pandemic period and from 3 to 3050 in the pandemic period.^9^, ^10^, ^21^, ^22^ The number of cervical treatments per month was reduced by −1.70 SD (95% CI −2.50 to −0.90, *p* < 0.001) (Figure 4B). The reported number of diagnoses of pre‐invasive lesions ranged from 89 to 1418 in the pre‐pandemic period and from 15 to 1257 in the pandemic period.^9^, ^10^, ^21^, ^22^ The number of pre‐invasive lesions diagnoses per month was reduced by −4.61 SD (95% CI −7.90 to −1.33, *p* = 0.006) (Figure 4C). The reported number of cervical cancer diagnoses ranged from 17 to 1955 in the pre‐pandemic period and from four to 1352 in the pandemic period.^1^, ^3^, ^10^, ^11^, ^18^, ^19^, ^20^, ^21^, ^22^ The number of invasive cancer diagnoses per month was reduced by −0.85 SD (95% CI −1.52 to −0.19, *p* = 0.012) (Figure 4D).

The statistical heterogeneity between studies was substantial for all outcomes (colposcopies: *I* ^2^ = 60.97%, *p* = 0.075; cervical treatments: *I* ^2^ = 52.92%, *p* = −0.081; pre‐invasive lesions diagnoses: *I* ^2^ = 92.45%, p < 0.001; cervical cancer diagnoses: *I* ^2^ = −71.07%, *p* = 0.002), suggesting differences in study characteristics, which may contribute to variations in outcomes.

**FIGURE 5:**
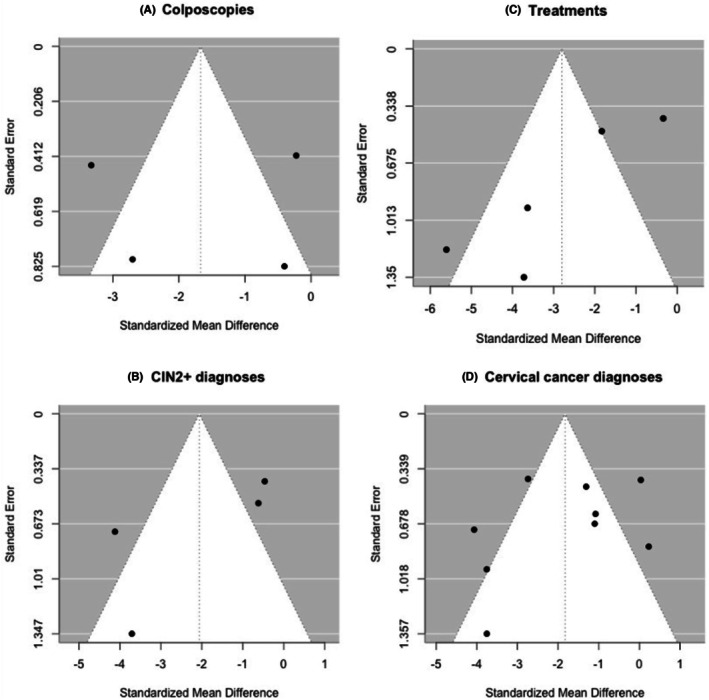
Funnel plot for review outcomes.

The Egger test was not significant for colposcopies (*b* = −0.62, 95% CI −4.52 to 3.28, *p* = 0.435), cervical treatments (*b* = 0.17, 95% CI −2.77 to 3.11, *p* = 0.148), pre‐invasive lesions diagnoses (*b* = 0.59, 95% CI −4.79 to 5.97, *p* = 0.108), and cervical cancer diagnoses (*b* = −0.47, 95% CI −2.44 to 1.51, *p* = 0.671), indicating no strong evidence of small‐study effects (Figure 5A–D).

Table 3 reports the SMD with 95% CI obtained from the meta‐regression for all outcomes with the variables “data source” and “screening coverage”, according to all combinations between moderators. Reductions in SMD were observed when data were from countries with screening coverage <70%, both in the case of data from institutional databases and registries. Reduction in pre‐invasive lesion diagnoses was less pronounced in the context of the institutional database. Regarding colposcopies and cervical cancer diagnoses, the moderators had no relevant impact, with SMD values remaining similar across combinations (Table 3).

**TABLE 3:** Meta‐regression for Standardized Mean Difference according to data source and screening coverage.

Outcome	Data source	Coverage	Standardized mean difference	95% CI	*p*
Colposcopies	Institution	<70%	−1.58	−2.52 to −0.64	0.001
	Institution	≥70%	−1.58	−2.52 to −0.64	0.001
	Registry	<70%	−1.41	−2.25 to −0.57	0.002
	Registry	≥70%	−1.41	−2.25 to −0.57	0.002
Cervical treatments	Institution	<70%	−1.80	−2.69 to −0.91	<0.001
	Institution	≥70%	−1.02	−1.83 to −0.22	0.013
	Registry	<70%	−2.46	−3.72 to −1.20	<0.001
	Registry	≥70%	−1.68	−2.56 to −0.79	<0.001
Pre‐invasive lesions diagnoses	Institution	<70%	−3.53	−4.89 to −2.17	<0.001
	Institution	≥70%	−1.45	−2.31 to −0.59	0.001
	Registry	<70%	−7.59	−10.06 to −5.11	<0.001
	Registry	≥70%	−5.52	−8.08 to −2.95	<0.001
Cervical cancers diagnoses	Institution	<70%	−0.73	−1.49 to 0.03	0.061
	Institution	≥70%	−1.07	−1.98 to −0.16	0.026
	Registry	<70%	−0.36	−1.94 to 1.22	0.650
	Registry	≥70%	−0.70	−1.99 to 0.59	0.284

The meta‐regression effectively explained all heterogeneity for cervical treatments and pre‐invasive lesions diagnoses (residual heterogeneity *I* ^2^ = 0.00% and *R* ^2^ = 100% for both outcomes, with *p* = 0.056 and *p* = 0.003, respectively), while leaving low (*I* ^2^ = 18.33%, *R* ^2^ = 34.29%, *p* = 0.040) and substantial (*I* ^2^ = 52.70%, *R* ^2^ = 27.64%, *p* = 0.002) residual heterogeneity for colposcopies and cervical cancer diagnoses, respectively. These findings suggest that additional, unmeasured factors may influence the variability in these outcomes, particularly for cervical cancer diagnoses.

## 4 DISCUSSION

The findings of this systematic review and meta‐analysis highlight the impact of the COVID‐19 pandemic on cervical cancer prevention, with reductions in colposcopies, cervical treatments, pre‐invasive lesion diagnoses, and cervical cancer diagnoses.

We observed substantial heterogeneity among the included studies, which can be attributed to variations in cervical cancer screening settings, including the type of screening test used, the presence or absence of a centralized call–recall system, and differences in healthcare systems, data sources, and screening coverage. These differences include, but are not limited to, the type of screening test used, ranging from visual inspection with acetic acid (VIA) in Ethiopia to cytology‐based screening in the USA, Canada, Brazil, and Romania, and HPV testing in England, Wales, Italy, and Portugal. Another key source of variation is the presence of systems overseeing the screening program, with established call–recall systems in place in countries such as England, Wales, Portugal, Italy, and Canada. In contrast, screening remains largely opportunistic in Brazil, Romania, and Ethiopia. Data from countries with low screening coverage were associated with greater reductions in cervical treatments and pre‐invasive lesion diagnoses. However, screening coverage may not be the sole contributing factor. As previously discussed, differences in healthcare settings could also play a role.

Regarding colposcopies and cervical cancer diagnoses, the moderators had no relevant impact, with SMD values remaining similar across combinations of data sources and screening coverage. Meta‐regression addressed heterogeneity for cervical treatments and pre‐invasive lesions diagnoses but left residual variability for colposcopies and cervical cancer diagnoses, suggesting other influencing factors such as differences in healthcare settings.

A key difference in cervical cancer screening settings is the use of the HPV test as the primary test. The HPV test has a higher sensitivity and negative predictive value than cytology and is usually performed at longer intervals (5 vs. 3 years).^23^ Those characteristics could prove helpful in reducing the impact of potential future global events, as the longer interval with fewer lifetime tests makes short‐term suspensions less critical, and the higher sensitivity increases the likelihood of lesion detection after screening resumption.

The role of the data source should also be acknowledged. Indeed, studies based on national or regional registries may provide more standardized data; however, potential delays in updates may underestimate the effect of short‐term disruptions. On the other hand, studies derived from institutional databases might be more sensitive in capturing acute changes, but at the cost of potential selection and reporting biases. Moreover, registry data are often derived from centers that primarily deal with primary screening tests or colposcopies and therefore may not have completely reliable data regarding treatments or diagnoses of invasive pathology.

The duration and modality of activity suspension are other factors to take into consideration, although it is difficult to quantify the exact duration for each country. In most cases, the declaration of the COVID‐19 pandemic led to a complete suspension of invitations for cervical screening, where call and recall systems were in place, as well as all non‐urgent clinical activities, including colposcopy and cervical treatments. This type of suspension has likely had the most significant impact, given that in late 2020 or early 2021, the suspension was partial, and most activities were maintained.

The results of this systematic review align with the available reports highlighting the substantial disruptions to cervical cancer prevention services during the COVID‐19 pandemic. In particular, our findings complete the systematic review of Ferrara et al. of 2022, who demonstrated reduced human papillomavirus vaccination, screening activities, diagnosis, and treatments of cervical cancers.^12^ Our work adds specific insights into colposcopy clinics' activities, emphasizing the reduction of colposcopies, diagnoses of pre‐invasive lesions, and treatments. These reductions are concerning given modeling studies predicting remarkable long‐term impacts; for instance, the study from Castanon et al. of 2021 reported that a 6‐month screening delay in England could lead to 630 additional cervical cancer cases over a 3 to 5‐year screening round with a progression rate over 6 months from untreated high‐grade CIN from 0.12 to 1.1% dependent on age.^24^, ^25^ Models evaluated by Burger et al. in 2022 projected a relative increase in symptomatically detected cancer cases during a 1‐year delay period between 38% and 170%, with higher figures for under‐screened patients whose last cytology screen was 5 years prior to the disruption period.^26^ Davies et al. suggested that due to the temporary cessation of screening during the pandemic, there would be 586, 228, and 105 extra cases of local cancer, those with regional disease, and those with distant disease throughout England over the next 3 years.^20^ The delay in prevention services, particularly for patients with known CIN or with symptoms suggestive of cervical cancer, may also lead to stage shift. Indeed, Popescu et al. reported that the percentage of late‐stage cervical cancers rose by 17% during the pandemic in Romania,^1^ and Bonadio et al. evidenced how cervical cancer patients had more advanced‐stage diseases in their first visit in comparison to a similar period before the pandemic in Brazil.^18^ Although it is tempting to quantify the impact of the pandemic on patients based on the presented data, it is not possible to draw firm conclusions, as the studies included did not report data separately for these groups.^1^, ^4^, ^5^, ^8^

Given that most national health authority guidelines allowed symptomatic patients to avoid delaying access to healthcare facilities, and that recommendations from scientific societies also reaffirmed this condition,^27^, ^28^ it can be assumed that the suspension of services may have less impacted this category of patients, but only if they were not afraid to visit healthcare facilities for fear of contagion.

The findings of this study underline the urgent need to strengthen global cervical cancer prevention programs to ensure resilience during future crises, like natural disasters or infectious disease outbreaks.^19^ Since high screening coverage demonstrated the capacity to mitigate service disruptions, it should be prioritized in healthcare policies.^9^ Expanding organized screening programs in low‐coverage areas could reduce disparities and improve outcomes. This is particularly relevant for lower income countries, which can benefit from coordinated community‐based approaches.^21^ Moreover, adaptive strategies, such as telemedicine, self‐sampling, use of mobile units, and prioritization of high‐risk cases (e.g., biomarkers positivity), may have a crucial role in maintaining essential services during emergencies.^2^, ^6^, ^8^, ^23^, ^26^, ^27^, ^28^, ^29^ While individuals with positive self‐samples still require follow‐up assessment in clinical settings, where fear of contagion may persist, self‐sampling can nonetheless help overcome the initial barrier to screening by allowing those with negative results to avoid healthcare facilities altogether.

Studies have shown that non‐attenders were disproportionately affected, with reduced access to opportunistic screening and a greater likelihood of delayed diagnosis due to service interruptions. Therefore, it should be further emphasized that greater efforts are needed to engage non‐attenders in cervical screening, as they appear to be the most vulnerable during healthcare disruptions such as the COVID‐19 pandemic.^24^, ^30^

In addition to the expected impact on cervical cancer diagnosis, the disruption of cervical cancer prevention services may have a non‐negligible impact on emotional well‐being. A patient survey conducted in 2020 in the UK highlighted how patients who have had their tests postponed due to pandemics report “anxiety” and “frustration” as the most common emotion, particularly regarding the feeling that COVID‐19 patients were prioritized over other patients.^31^

Future research should focus on evaluating the long‐term impact of these disruptions on cervical cancer incidence and mortality, using real‐world data. Comparative studies across healthcare systems could provide further insights into effective strategies to ensure continuity of care during global health crises.

The major strength of this systematic review is the inclusion of studies from different world regions and healthcare settings, enhancing the generalizability of the results. Evaluating the effect of moderators by meta‐regression further deepened the analysis, allowing the identification of factors that may reduce or increase the volume of prevention services. However, some limitations should be acknowledged. The residual heterogeneity after meta‐regression for the outcomes “colposcopies” and “cervical cancer diagnoses” suggests the potential role of unmeasured factors, such as differences in healthcare resource allocation or pandemic response between different countries. Including data from retrospective studies may introduce bias, and the small number of included studies may limit statistical power.

## 5 CONCLUSION

This study highlights the disruptions to cervical cancer prevention services during the COVID‐19 pandemic, with marked reductions in colposcopies, cervical treatments, diagnoses of pre‐invasive lesions, and diagnoses of cervical cancers. A screening coverage below 70% exacerbated these declines, although it is unlikely to be the sole contributing factor. Strong organizational and resource availability may shape healthcare service delivery during crises. Strengthening cervical cancer prevention programs through organized screening, resilient infrastructures, and adaptive strategies will be crucial to safeguard cancer prevention during future global health emergencies.

## AUTHOR CONTRIBUTIONS

Giovanni Delli Carpini: conceptualization, data curation, formal analysis, writing—original draft. Zahid Mammadov: data curation, formal analysis, writing—original draft. Simon Leeson: conceptualization, formal analysis, writing—review and editing. Anne Hammer: methodology, writing—review and editing. Mihaela Grigore: methodology, writing—review and editing. Andrea Ciavattini: conceptualization, supervision, writing—review and editing.

## FUNDING INFORMATION

No financial support was received.

## CONFLICT OF INTEREST STATEMENT

A.H. has received reagents for free from Roche Diagnostics, Denmark, outside the submitted work, and reports receiving a consulting fee from Exeltis, Norway. The remaining authors declare no conflict of interest.

## Supporting information


**File S1.** Complete search strategy.

## Data Availability

Original data are available from the corresponding author upon reasonable request.
