# Supplementary material for: The effect of healthcare disruptions during the COVID‐19 pandemic on colposcopy services and practice: A systematic review and meta‐analysis
Source: Acta Obstet Gynecol Scand. 2025 Oct 8;104(12):2215–25. doi: 10.1111/aogs.70066 (PMC12668810; doi:10.1111/aogs.70066)
Supplement: Supplementary file 1 — File S1. Complete search strategy. [file AOGS-104-2215-s001.pdf]

Database: Ovid MEDLINE(R) ALL
Search Strategy:

1 COVID-19/ (122833)
2 (2019 novel coronavirus disease or 2019 novel coronavirus infection or 2019 ncov disease or 2019 ncov infection or 2019-ncov disease or 2019-ncov diseases or 2019-ncov infection or 2019-ncov infections or covid 19 or covid 19 pandemic or covid 19 virus disease or covid 19 virus infection or covid-19 or covid-19 pandemic or covid-19 pandemics or covid-19 virus disease or covid-19 virus diseases or covid-19 virus infection or covid-19 virus infections or covid19 or coronavirus disease 19 or coronavirus disease 2019 or coronavirus disease-19 or disease 2019, coronavirus or disease, 2019-ncov or disease, covid-19 virus or infection, 2019-ncov or infection, covid-19 virus or infection, sars-cov-2 or pandemic, covid-19 or sars cov 2 infection or sars coronavirus 2 infection or sars-cov-2 infection or sars-cov-2 infections or virus disease, covid-19 or virus infection, covid-19).mp. (199848)

3 exp *COVID-19/ or COVID-19.mp. (197441)
4 exp *Uterine Cervical Dysplasia/ or Uterine Cervical Dysplasia.mp. (4466)
5 (Cervical intraepithelial neoplasia grade 2 or Cervical Intraepithelial Neoplasia Grade 2 3).mp. (556)
6 (cervical pre-cancer* or cervical pre-cancer*).mp. [mp=title, abstract, original title, name of substance word, subject heading word, floating sub-heading word, keyword heading word, organism supplementary concept word, protocol supplementary concept word, rare disease supplementary concept word, unique identifier, synonyms] (148)
7 (precancerous cervix or pre-cancerous cervix).mp. [mp=title, abstract, original title, name of substance word, subject heading word, floating sub-heading word, keyword heading word, organism supplementary concept word, protocol supplementary concept word, rare disease supplementary concept word, unique identifier, synonyms] (11)
8 (CIN 2 or CIN II or CIN 3 or CIN III).mp. [mp=title, abstract, original title, name of substance word, subject heading word, floating sub-heading word, keyword heading word, organism supplementary concept word, protocol supplementary concept word, rare disease supplementary concept word, unique identifier, synonyms] (3198)
9 cervical dysplasia.mp. (4919)
10 Cervical Intraepithelial Neoplasia/ or Uterine Cervical Neoplasms/ (80896)
11 exp *Adenocarcinoma/ or Adenocarcinoma.mp. or "AIS".mp. [mp=title, abstract, original title, name of substance word, subject heading word, floating sub-heading word, keyword heading word, organism supplementary concept word, protocol supplementary concept word, rare disease supplementary concept word, unique identifier, synonyms] (442928)
12 1 or 2 or 3 (199848)
13 4 or 5 or 6 or 7 or 8 or 9 or 10 or 11 (517311)

14 screen*.mp. [mp=title, abstract, original title, name of substance word, subject heading word, floating sub-heading word, keyword heading word, organism supplementary concept word, protocol supplementary concept word, rare disease supplementary concept word, unique identifier, synonyms] (928648)

15 screening.mp. [mp=title, abstract, original title, name of substance word, subject heading word, floating sub-heading word, keyword heading word, organism supplementary concept word, protocol supplementary concept word, rare disease supplementary concept word, unique identifier, synonyms] (676625)

16 (Cancer Screening or Cancer Screening Test or Cancer Screening Tests or Test, Cancer Screening or Tests, Cancer Screening).mp. (35848)

17 14 or 15 or 16 (928648)

18 12 and 13 and 17 (79)

19 limit 18 to yr="2018 -Current" (79)

20 limit 19 to english language (77)

21 limit 20 to covid-19 (77)

22 limit 21 to (female and "all adult (19 plus years)") (23)

23 (cervical smear or cervical smears or smear, cervical or smear, vaginal or smears, cervical or smears, vaginal or vaginal smear or vaginal smears or (cancer screening or cancer screening test or cancer screening tests or screening, cancer or screening test, cancer or screening tests, cancer or test, cancer screening or tests, cancer screening)).mp. (57756)

24 17 or 23 (944319)

25 12 and 13 and 24 (79)

26 limit 25 to (english language and yr="2020") (13)

27 exp *Vaginal Smears/ (10534)

28 23 or 27 (57756)

29 12 and 13 and 28 (38)

30 limit 29 to yr="2020 -Current" (38)
